# Molecular piracy in deep-sea hydrothermal vent: phage-plasmid interactions revealed by phage-FISH in *Marinitoga piezophila*

**DOI:** 10.1128/aem.02306-24

**Published:** 2025-02-27

**Authors:** Min Jin, Ouafae Rouxel, Nadège Quintin, Claire Geslin

**Affiliations:** 1 Univ Brest, Ifremer, BEEP 27002 , Plouzané, France; 2 State Key Laboratory Breeding Base of Marine Genetic Resource, Third Institute of Oceanography, Ministry of Natural Resources 118477 , Xiamen, China; 3 LIA/IRP 1211 MicrobSea, Sino-French International Laboratory of Deep-Sea Microbiology, Plouzané, France; Colorado School of Mines, Golden, Colorado, USA

**Keywords:** deep-sea hydrothermal vents, mobile genetic element, phage, *Marinitoga piezophila*, phage-FISH, molecular piracy, horizontal gene transfer

## Abstract

Prokaryotes and mobile genetic elements (MGEs, such as viruses and plasmids) interact extensively, leading to horizontal gene transfer (HGT) and consequent microbial evolution and diversity. However, our knowledge of the interactions between MGEs in deep-sea hydrothermal ecosystems is limited. In this study, we adapted a phage-fluorescence *in situ* hybridization (phage-FISH) approach to visualize and quantify the dynamics of phage–plasmid interactions in an anaerobic, thermophilic deep-sea bacterium, *Marinitoga piezophila*. Notably, our results revealed that plasmid signals were detected in viral particles released from lysed cells, indicating that mitomycin C not only induced plasmid replication but also its packaging into phage particles. Further analysis of the DNA content in purified virions showed that the phage capsids incorporated plasmid DNA even without induction, and the majority of capsids (up to 70%) preferentially packaged plasmid DNA rather than viral DNA after induction. Therefore, this study provided direct evidence of molecular piracy in the deep-sea hydrothermal ecosystem, highlighting the important roles of selfish MGEs in virus–host interactions and HGT in extreme marine environments.

**IMPORTANCE:**

Deep-sea hydrothermal vents are hotspots for microbes. Several studies revealed that virus-mediated horizontal gene transfer (HGT) in deep-sea hydrothermal vent ecosystems may be crucial to the survival and stability of prokaryotes in these extreme environments. However, little is known about the interaction between viruses and other mobile genetic elements (MGEs, such as plasmids), and how their interactions influence virus-mediated HGT in these ecosystems. In this study, we adapted a phage-fluorescence *in situ* hybridization approach to directly monitor the dynamics of phage–plasmid–host interactions at the single-cell level in the *Marinitoga piezophila* model. Interestingly, our results indicate that plasmid DNA could not only be induced by mitomycin C to a great extent but also hijacked viral assembly machinery to facilitate its propagation and spread. Therefore, the data presented here imply that the interaction between the viruses and other MGEs could play profound roles in virus–host interaction and virus-mediated HGT in the deep-sea hydrothermal ecosystem.

## INTRODUCTION

Deep-sea hydrothermal vents are one of the most extreme environments on Earth and are characterized by steep physicochemical gradients, high hydrostatic pressure, high temperatures, obscurity, and prevalence of chemosynthesis (1). The steep physicochemical gradients in deep-sea hydrothermal vents generate ecological niches and habitats for a vast diversity of mesophilic and (hyper-)thermophilic *Bacteria* and *Archaea*, making deep-sea hydrothermal vents biodiversity hotspots in the deep sea (1, 2). Over the past decades, significant efforts have been made to understand the diversity, metabolic capacity, physiological adaptations, and ecology of prokaryotes inhabiting hydrothermal vents (1, 3–6). These studies have reported that the prokaryotes in vent ecosystems are highly diverse and play important roles in biomass production and biogeochemical cycles on a global scale.

Prokaryotic viruses have been found to play a major role in the evolution and diversity of microbial communities, as well as in global biochemical cycles in marine environments (7–10). However, viral communities in deep-sea hydrothermal ecosystems have received much less research attention compared to prokaryotes. Virus-like particles (VLPs) are more abundant than prokaryotes in these ecosystems (11) and infect a wide diversity of microbial hosts (12, 13). Some studies found that the abundance of planktonic viruses in hydrothermal water samples can range from 10^5^ to 10^6^ VLPs/mL, which is much higher than their abundance in the surrounding seawater samples (14, 15). A comparative study on viral diversity across different vent ecosystems showed that hydrothermal vent viruses are largely endemic to individual vent sites, indicating restricted dispersal (16). Additionally, previous research revealed that these ecosystems harbor abundant lysogenic populations and temperate viruses, suggesting that virus-mediated HGT may be crucial to the survival and stability of prokaryotes in extreme environments (12). Several metagenomic studies identified various auxiliary metabolic genes in deep-sea hydrothermal viruses, including those involved in the metabolism of carbohydrates, amino acids, methane, sulfur, cofactors, and vitamins (17, 18), which are thought to compensate for host metabolism and participate in biogeochemical cycles (11). However, a large fraction of viral sequences obtained from high-throughput sequencing lacks similarity with known sequences in the database (19, 20). Moreover, metagenomic data do not provide in-depth information on the interactions between a specific virus-host pair (20, 21). Therefore, to understand the role of viruses in deep-sea ecosystems, it is crucial to isolate new viruses and characterize their interactions with their hosts.

To date, only 12 viruses have been isolated and characterized from deep-sea vents, of which 3 are archaeoviruses and 9 are bacterioviruses. In a previous study, we isolated and characterized MPV1, which infects *Marinitoga piezophila*, an anaerobic, piezophilic, thermophilic, and chemo-organotrophic sulfur-reducing bacterium of the order *Petrotogales* (22). Interestingly, we found that mitomycin C treatment induced the replication of MPV1 prophage, as well as pMP1, a 13.3 kb plasmid of *M. piezophila*. Quantitative polymerase chain reaction analysis showed that the relative concentration of plasmid DNA was 10-fold higher than viral DNA after induction. Moreover, DNA sequencing analysis revealed a 20-fold higher coverage of plasmid reads compared to “true viral” reads in purified virions after induction. The pMP1/MPV1 system represents an example of molecular piracy. The term “molecular piracy” has been coined to describe a biological phenomenon in which one replicon (the pirate) uses the structural proteins encoded by another replicon (the viral helper) to package its own genome and thus allow the pirate’s propagation (23). The type of relationship between a virus and another MGE within the same host has been described in organisms from all three domains of life (24–26). Thus, our previous study on the pMP1/MPV1 system highlights the potential role of selfish MGEs in facilitating HGT in the deep-sea hydrothermal ecosystem (22). However, more in-depth analysis is required to improve our understanding of the phage–plasmid interactions, especially at the single-cell level. Allers et al. developed a phage-fluorescence *in situ* hybridization (FISH) method, which can be used to detect and visualize intra- and extracellular phage DNA at the single-cell level and simultaneously quantify the infection status of a single cell (27). In this study, we used a modified phage-FISH method to visualize both phage and plasmid DNA in the extremophilic model *M. piezophila* and directly monitor the dynamics of phage–plasmid–host interactions at the single-cell level.

## MATERIALS AND METHODS

### Bacterial strain, phage, and culture conditions

*M. piezophila* KA3 (UBOCC-M-2489) was first isolated from a deep-sea hydrothermal chimney sample collected at a depth of 2,630 m on the East-Pacific Rise (12°48721′N, 103°56351′W) (28). The optimal pressure and temperature for its growth are 40 MPa and 65°C, respectively; however, after several subcultures at 0.1 MPa, the *M. piezophila* KA3 cells have been adapted to atmospheric pressure. The optimal doubling time (at 65°C and 40 MPa) of *M. piezophila* KA3 is 21 min. In this study, the *M. piezophila* KA3 cells were anaerobically cultured at 0.1 MPa and 65°C in a modified Ravot medium (per 1 L medium: 0.3 g NH_4_Cl, 0.5 g MgCl_2_·6H_2_O, 0.1 g CaCl_2_·2H_2_O, 0.5 g KCl, 0.83 g NaCH_3_COO·3H_2_0, 2 g yeast extract, 2 g tryptone, 30 g sea salt, 3.3 g piperazine-*N*,*N*-bis(2-ethanesulfonic acid), 2 g maltose, 0.001 g resazurin, 0.35 g KH_2_PO_4,_ 0.35 g K_2_HPO_4_, 10 g elemental sulfur, and 1 mL polyvitamins solution, pH 6.0). The medium was sparged with 100% N_2_ gas to make it anoxic. Additionally, it was reduced by adding 0.05% (wt/vol) Na_2_S·9H_2_O. MPV1 is a temperate siphophage isolated from the KA3 strain by mitomycin C induction (22). It contains a dsDNA genome of 43.7 kb in length. KA3 cells also carry a 13.3 kb plasmid (pMP1). pMP1 contains 13 ORFs, and no known structural genes involved in virion formation were identified. All seven functionally annotated genes of pMP1 were assigned to gene families involved in DNA interactions (i.e., transcription, regulation, and DNA metabolic processes) (22).

### Digoxigenin (DIG) probe design and synthesis

Several 300 bp dsDNA probes were synthesized for the detection of phage and plasmid DNA. The probes were subjected to BlastN to ensure that they had no significant similarity (<60% identity) with other known DNA sequences in the *M. piezophila–*MPV1–pMP1 model. Additionally, a 300 bp dsDNA probe targeting the *Thermococcus* genome was included as the negative control (NC). The NC probe had no significant similarity (<60% identity) with any known DNA sequences in the *M. piezophila–*MPV1–pMP1 model. The primer sequence and the target region of each probe are provided in Table S1. The DIG-labeled probes were synthesized by incorporating DIG-dUTP into the dsDNA molecules using the PCR DIG Probe Synthesis Kit (Roche, Swiss), according to the manufacturer’s instructions. The PCR products were then column purified and assessed by 3% agarose gel electrophoresis to verify their size and DIG incorporation. The concentration of the purified DIG probes was determined using a nanodrop spectrometer (Thermo Scientific, USA) and was then adjusted to 5 ng/µL. The DIG probes were stored at −20°C until further use.

### FISH assay of *M. piezophila* cells

*M. piezophila* KA3 cells in the early-log phase were divided into two treatment groups, namely mitomycin C-induced and uninduced groups. The induced group was incubated with 5 µg/mL mitomycin C for 2 h, while the uninduced group was cultured for 2 h without any treatment. Both the cultures were harvested by centrifugation at 4,000 rpm for 15 min, resuspended in phosphate-buffered saline (PBS), and fixed with 2% formaldehyde (methanol-free) for 1.5 h at 4°C. The cells were then washed two times with PBS and resuspended in 1:1 (vol/vol) PBS and ethanol. FISH of the induced and uninduced cells was conducted using a modified geneFISH protocol (27, 29). Briefly, the fixed cells were spotted on the slides and air-dried at 37°C. Thereafter, the cells were washed several times with 1× PBS and water and permeabilized for 1 h at 37°C using a permeabilization solution (0.5 mg/mL lysozyme, 1× PBS [pH 7.4], 0.1 M Tris–HCl [pH 8.0], and 0.05 M EDTA). Subsequently, the cells were washed with 1× PBS and water and incubated with 0.01 M HCl for 10 min to inactivate their endogenous peroxidases. The cells were washed again with 1× PBS and ethanol, air-dried, and incubated with horseradish peroxidase (HRP)-labeled 16S rRNA probes (EUB338 [30]) in the rRNA hybridization buffer (35% formamide, 10% Dextran sulfate [DS], 0.9 M NaCl, 20 mM Tris–HCl [pH 8.0], 1% nucleic acid blocking reagent, 0.25 mg/mL sheared salmon sperm DNA, 0.25 mg/mL yeast RNA, and 0.02% SDS) at 46°C for 3 h. The cells were then washed with an rRNA washing buffer (70 mM NaCl, 5 mM EDTA pH 8.0, 20 mM Tris–HCl, and 0.01% SDS) for 15 min at 48°C, followed by 1× PBS for 10 min. Catalyzed reporter deposition (CARD)-FISH was performed by incubating the cells with rRNA–CARD buffer–Alexa488 tyramide mix (0.33 µg/mL Alexa488 and 0.0015% H_2_O_2_) at 37°C for 15 min. The cells were washed sequentially with 1× PBS, water, and ethanol and air-dried. Thereafter, the cells were treated with 150 µg/mL RNase A at 37°C for 1 h and washed with 1× PBS and water to digest the RNA. The cells were incubated with 0.2 M HCl for 10 min to inactivate the HRP; sequentially washed with 1× PBS, water, and ethanol; and air-dried. Subsequently, for the gene hybridization step, the cells were placed in a gene hybridization buffer (35% formamide, 5× Saline Sodium Citrate [SSC] buffer, 10% DS, 0.1% SDS, 20 mM EDTA, 1% nucleic acid blocking reagent, 0.25 mg/mL sheared salmon sperm DNA, and 0.25 mg/mL yeast RNA) at 46°C for 30 min and incubated with DIG phage or plasmid probes (5 pg/µL each probe in the gene hybridization buffer). The cells were then denatured at 85°C for 30 min and subjected to gene hybridization at 46°C for 3 h. Subsequently, the cells were successively washed with gene washing buffer I (2× SSC and 0.1% SDS), gene washing buffer II (0.1× SSC and 0.1% SDS), and 1× PBS. The cells were incubated in antibody-blocking solution (1× PBS and 1% Western Blocking Reagent) for 30 min and antibody-binding solution (1× PBS, 1% Western Blocking Reagent, and 0.3 U/mL anti-DIG HRP-conjugated antibodies) for 1.5 h. After several washes with antibody-blocking solution, the cells were incubated with gene CARD amplification buffer–Alexa594 tyramide mix (1× PBS, 20% DS, 0.1% blocking reagent, 2 M NaCl, and 0.33 µg/mL Alexa594) at 37°C for 45 min. The cells were then washed with 1× PBS, water, and ethanol and air-dried. Finally, the slides were mounted with SlowFade Gold antifade reagent and sealed with nail polish.

### FISH assay of purified phage particles

An early-log phase *M. piezophila* KA3 cell culture (500 mL) was incubated with 5 µg/mL mitomycin C for 2 h and centrifuged at 7,500 g and 4°C for 15 min to remove the debris and host cells (pellet). The supernatant was ultracentrifuged at 41,000 rpm and 10°C for 1.5 h using the Beckman Optima LE-80 K 45Ti rotor. The phage pellet was resuspended in 150 µL SM buffer (10 mM Tris-HCl, 100 mM NaCl, 5 mM CaCl_2_, and 20 mM MgCl_2_) and filtered through the Acrodisc Syringe Filter (0.45 µm) with HT Tuffryn Membrane. To prevent cellular DNA contamination, the purified phage samples were treated with 50 µg/mL DNase I at 37°C for 1 h. FISH of purified phage particles was performed as described previously (27). Briefly, phage particle suspension (50 µL) was spotted on glass slides, air-dried at 37°C for 1 h, and washed multiple times with water and ethanol. After air-drying, the phage particles were fixed with 1% paraformaldehyde at room temperature and sequentially washed with 1× PBS, water, and ethanol. After air-drying, the slides were incubated with 0.01 M HCl for 10 min and 0.2 M HCl for 10 min to lyse the phage capsids. The slides were washed with 1× PBS, water, and ethanol and air-dried. Finally, the slides were subjected to the subsequent FISH protocols, as described above, starting from “gene hybridization.”

### Microscopy, signal count, and statistical analysis

The hybridized slides were observed under a Zeiss Imager Z2 microscope equipped with the Apotome.2 sliding module and Colibri 7 light technology (Zeiss, Oberkochen, Germany). The Alexa488 and Alexa594 filter sets were used to obtain images of the 16S rRNA signals and the phage or plasmid gene signals, respectively. The micrographs were analyzed using the Zen software (Zeiss, Oberkochen, Germany). Signal counting was performed on images captured with the same exposure times. The cells in the Alexa488 channel and the phage or plasmid gene signals in the Alexa594 channel were manually marked and counted. The results were represented as the mean ± standard deviation (SD) of three replicates. Statistical significance between groups was analyzed using SPSS v11.5 (SPSS Institute, USA) based on Student’s *t* test. The *P* values of <0.05 and <0.01 were regarded as statistically significant (*) and highly statistically significant (**), respectively.

## RESULTS

### Design and synthesis of DIG probes for establishing FISH approaches in *M. piezophila*–MPV1–pMP1 model

To explore the interactions between *M. piezophila* cells, pMP1, and MPV1, we monitored the phage and plasmid DNA in *M. piezophila* cells and extracellular phage particles under an epifluorescence microscope using the modified phage-FISH method (27, 29). For the modified phage-FISH method, we designed and synthesized 300 bp DIG-labeled phage probes and plasmid probes that could detect their DNA. Multiple DIG-labeled probes were simultaneously used for target binding to increase the detection efficiency of FISH (27). Phage and plasmid probes were synthesized against five 300 bp target regions in the phage genome and four 300 bp target regions in the plasmid genome, respectively (Table S1). To prevent non-specific binding during FISH, these probes were validated to have no significant similarity (<60% identity) with other known DNA sequences in the *M. piezophila–*MPV1–pMP1 model. PCR was conducted to synthesize the DIG probes using DIG-labeled dUTP as the substrate. As shown in Fig. S1a and b, the length of the PCR products for four phages (1, 3, 4, and 5) and four plasmid probes were slightly increased along with the final concentration of DIG, suggesting the successful incorporation of DIG into these probes. In total, we synthesized four DIG-labeled phage and plasmid probes each. Additionally, we synthesized a 300 bp DIG-labeled NC probe against *Thermococcus*, which showed no significant similarity (<60% identity) with any known DNA sequences in the *M. piezophila–*MPV1–pMP1 model (Fig. S1c).

### Effects of mitomycin C induction on phage and plasmid replication in *M. piezophila* cells

**Fig 1:**
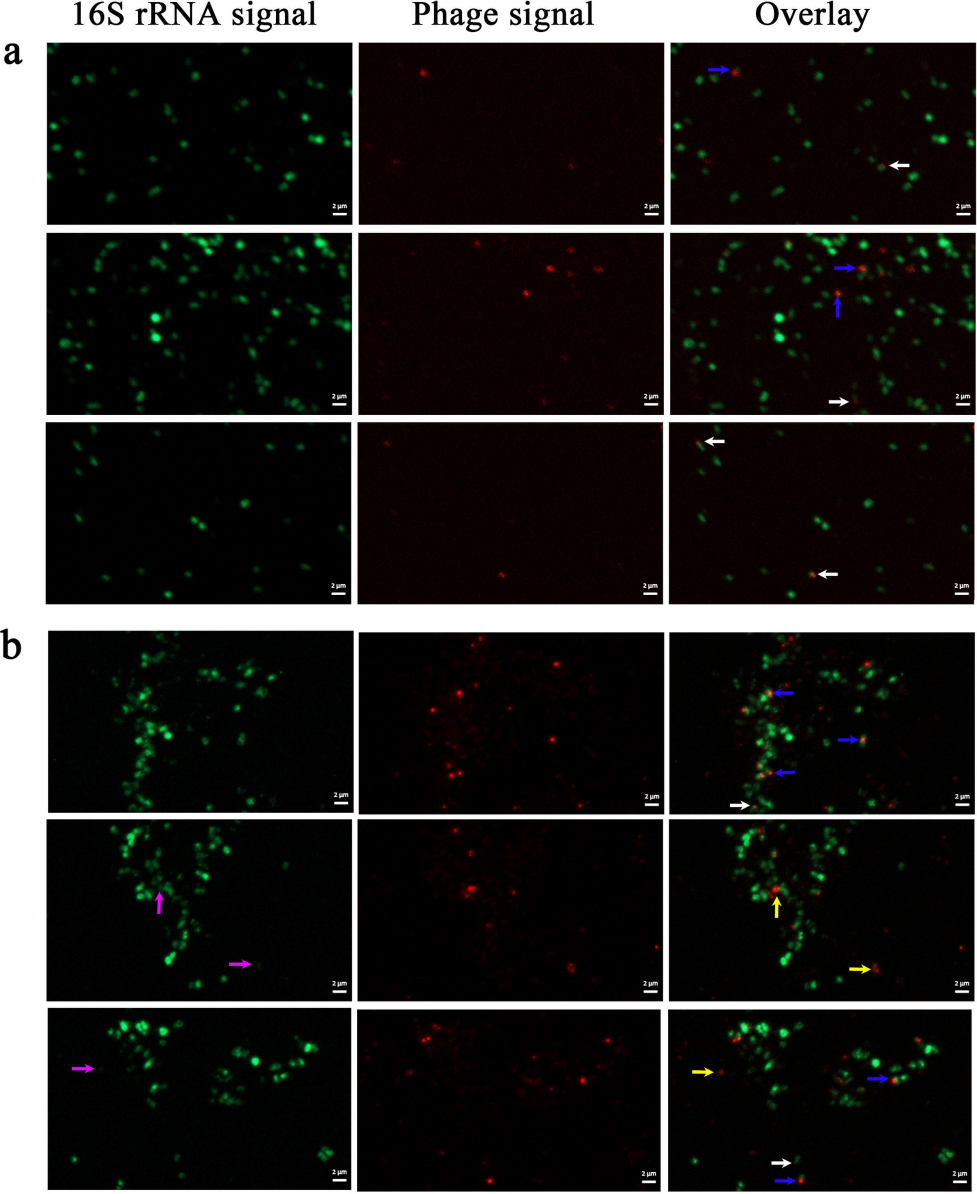
FISH of uninduced (**a**) and induced (**b**) *M. piezophila* cells using 16S rRNA probes (green) and phage probes (red). The images show representative epifluorescence micrographs after FISH. Left: 16S rRNA signal; center: phage signal; and right: overlay of 16S rRNA and phage signals. White arrows indicate early phage infections, blue arrows indicate advanced infections, yellow arrows indicate phages released from lysed cells, and purple arrows indicate lysed cells. Scale bars: 2 µm.

**Fig 2:**
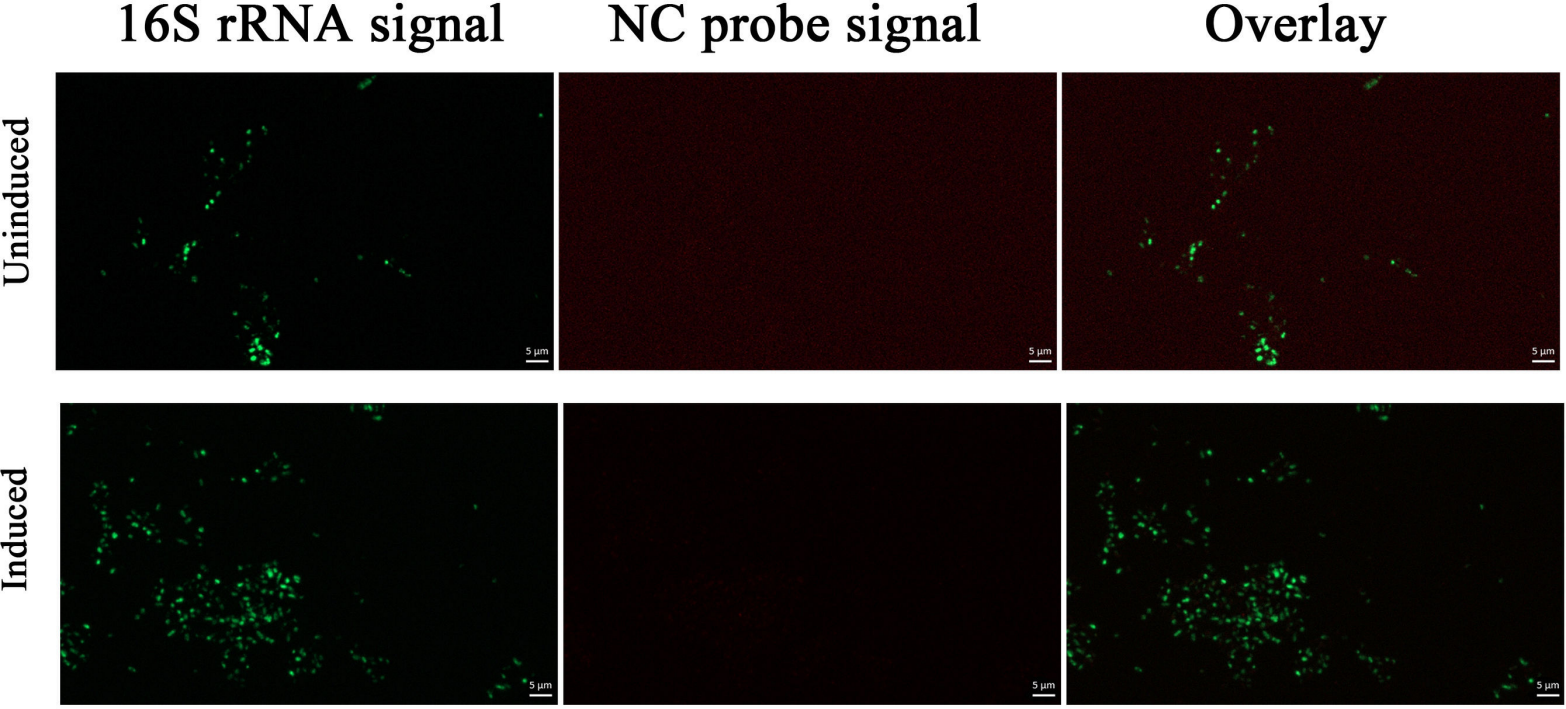
FISH of uninduced and induced *M. piezophila* cells using 16S rRNA probes (green) and negative control (NC) probes (red). The images show representative epifluorescence micrographs after FISH. Left: 16S rRNA signal; center: NC signal; and right: overlay of 16S rRNA and NC signals. Scale bars: 5 µm.

**Fig 3:**
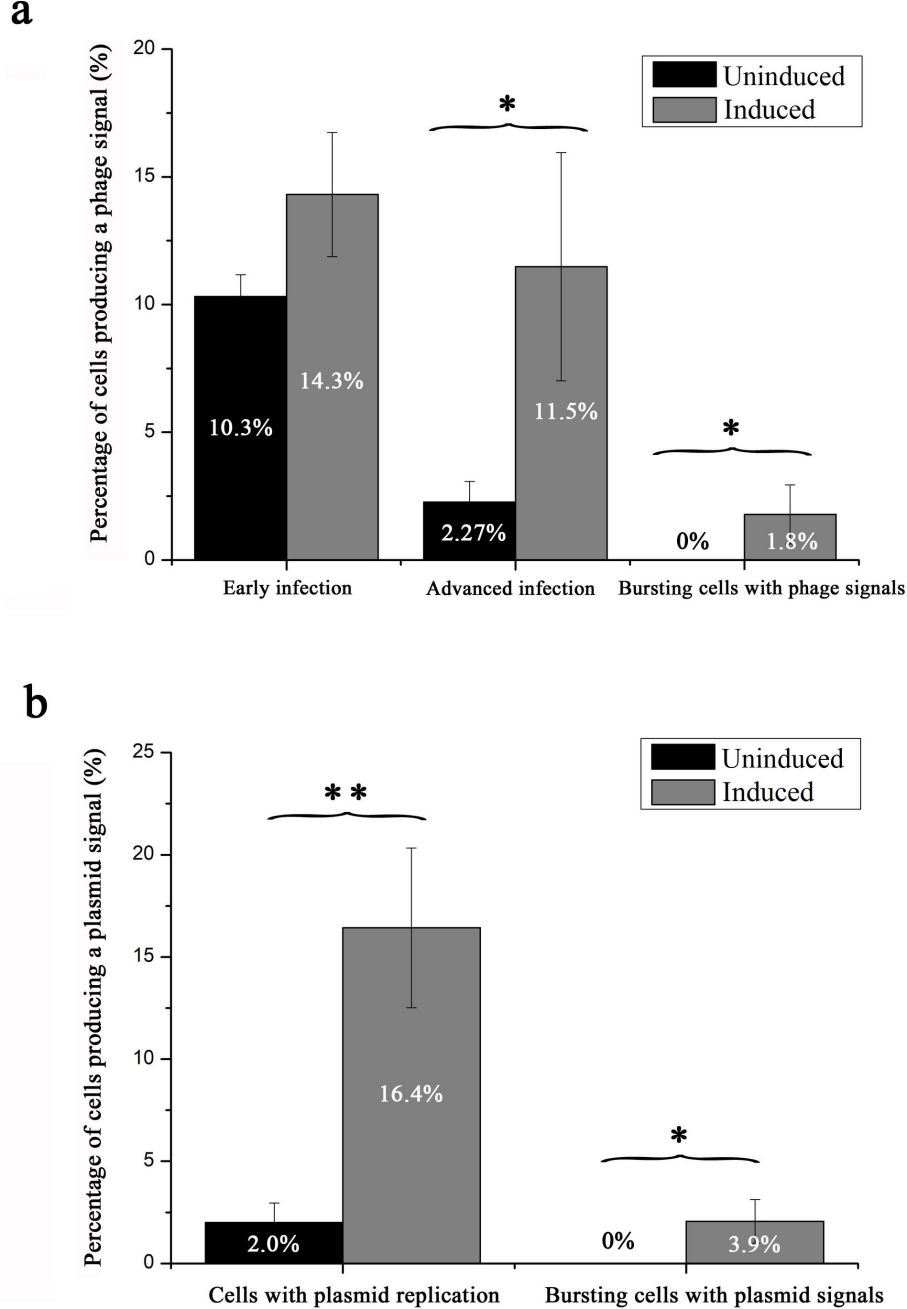
Effects of induction on phage (**a**) and plasmid replication (**b**) in *M. piezophila* cells as determined by FISH. The results are expressed as the means ± SD of three replicates. Pairwise groups that are significantly different are marked with asterisks (*P < 0.05 and **P < 0.01).

To investigate the effects of mitomycin C induction on phage infection in *M. piezophila* cells, FISH was performed on induced and uninduced *M. piezophila* cells using 16S rRNA and phage probes. As shown in Fig. 1, hybridized 16S rRNA probes emitted bright green signals corresponding to the 16S rRNA in the cytoplasm, while the hybridized phage probes emitted either a dot-like signal (indicated by white arrows) or a larger wide-spread signal (indicated by blue arrows) in the *M. piezophila* cells. The NC probes hybridized with <1% of the induced and uninduced cells, emitting dot-like signals (false positives) with the lowest size (Fig. 2) Allers et al. found that the signal size is correlated with the target number and indicated that the size of the phage signal could provide a semi-quantitative metric to describe the infection stage per cell (27). Therefore, based on the intracellular phage signal patterns in the *M. piezophila* model, the dot-like phage signal most likely represents an early phage infection, while the large widespread signal represents an advanced phage infection (late replication and assembly). Additionally, phage-FISH detected released phage particles (indicated by yellow arrows) from lysed *M. piezophila* cells with almost no 16S rRNA signal (indicated by purple arrows) (Fig. 1). In the uninduced group, only 12.6% of the cells emitted a phage signal (Fig. 1a and Fig. 3a), of which the majority (10.3%) emitted dot-like signals, indicating early infection. Moreover, the uninduced cells showed no cell lysis-like events (Fig. 1a and Fig. 3a). The phage signals detected in the uninduced group could likely correspond to the spontaneous induction of prophages. Meanwhile, approximately 27.6% of the induced *M. piezophila* cells emitted phage signals, indicating significantly higher phage replication in the induced cells compared to the uninduced cells (*P* < 0.05). Moreover, compared to the uninduced cells, significantly larger fractions of signal-emitting cells entered the advanced (11.5%, *P* < 0.05) and lysis stages (1.8%, *P* < 0.05) after induction (Fig. 1b and Fig. 3a). These results indicate that mitomycin C can induce MPV1 prophage to mediate massive lytic infection of *M. piezophila* cells.

**Fig 4:**
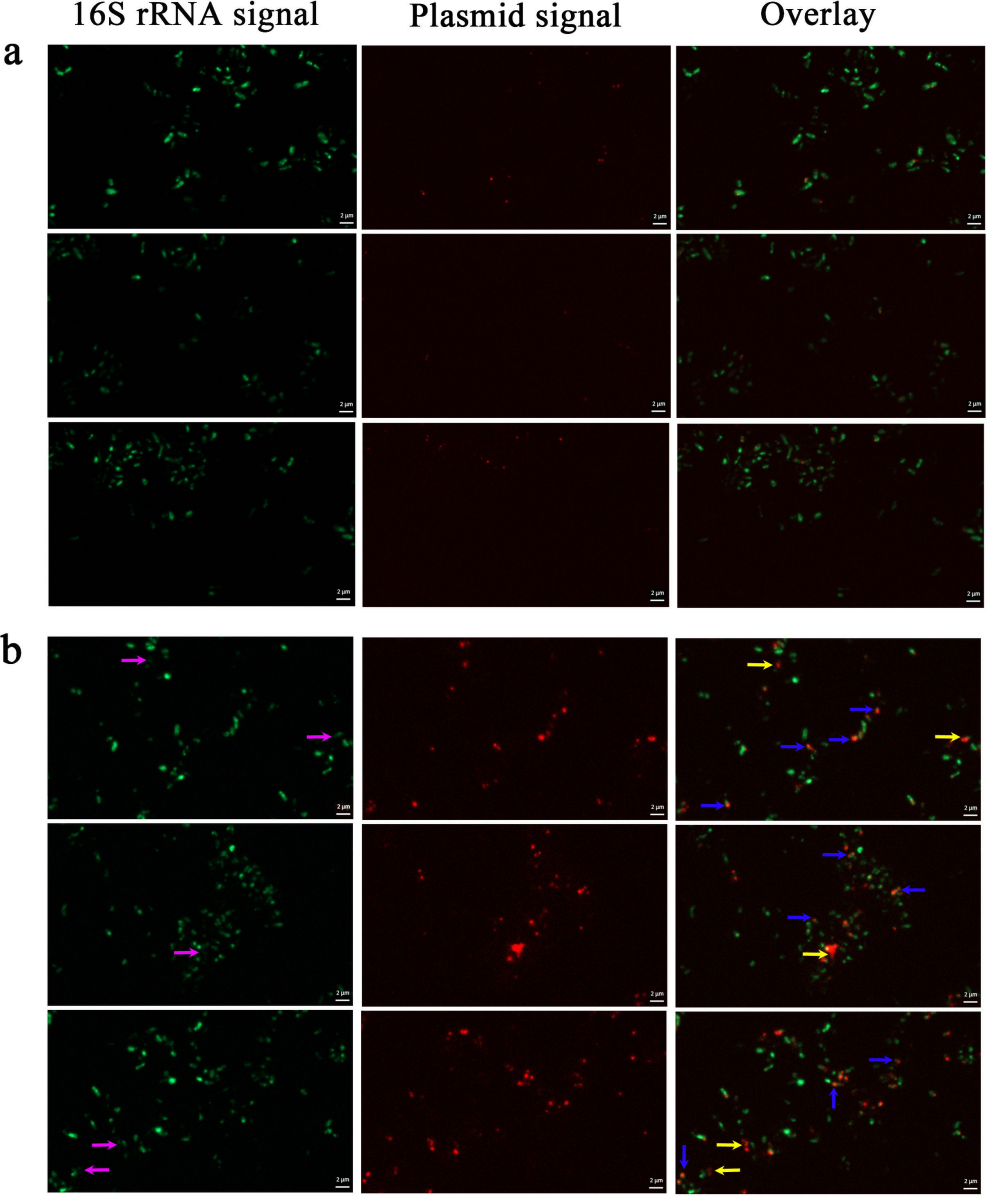
FISH of uninduced (**a**) and induced (**b**) *M. piezophila* cells using 16S rRNA probes (green) and plasmid probes (red). The images show representative epifluorescence micrographs after FISH. Left: 16S rRNA signal; center: plasmid signal; and right: overlay of 16S rRNA and plasmid signals. Blue arrows indicate induced plasmid replication, yellow arrows indicate virions containing plasmids released from lysed cells, and purple arrows indicate lysed cells. The first and third epifluorescence micrographs in (B) have some overlap, since they are adjacent fields of view for the same sample. Scale bars: 2 µm.

To investigate the effects of mitomycin C induction on plasmid replication in *M. piezophila* cells, FISH was performed on induced and uninduced *M. piezophila* KA3 cells using 16S rRNA and plasmid probes. The results showed that the hybridized plasmid probes emitted a dot-like signal in the uninduced cells (Fig. 4a) and a larger wide-spread signal in the induced cells (Fig. 4b, indicated by blue arrows). Based on the relationship between signal size and target number, the dot-like plasmid signal likely represents spontaneous plasmid replication with low copy number, while the large widespread plasmid signal likely represents the massive replication of plasmid after induction. Overall, the uninduced group only had numerous intracellular plasmid signals (2.0% of total cells) with the lowest size class corresponding to spontaneous plasmid replication, and almost no massive plasmid replication signal was visible (Fig. 4a and 3b). Compared to the uninduced *M. piezophila* cells, induced *M. piezophila* have significantly higher percentage of cells emitting plasmid replication signals (16.4%, *P* < 0.05), of which the majority emitted large wide-spread signals (Fig. 4b and 3b). Notably, 3.9% of plasmid signals were also associated with extracellular phage particles (indicated by yellow arrows) released from the lysed *M. piezophila* cells (Fig. 4b and 3b). These results indicate that mitomycin C can induce plasmid DNA replication, as well as its packaging in phage particles.

### Effects of mitomycin C induction on the DNA content of phage particles

**Fig 5:**
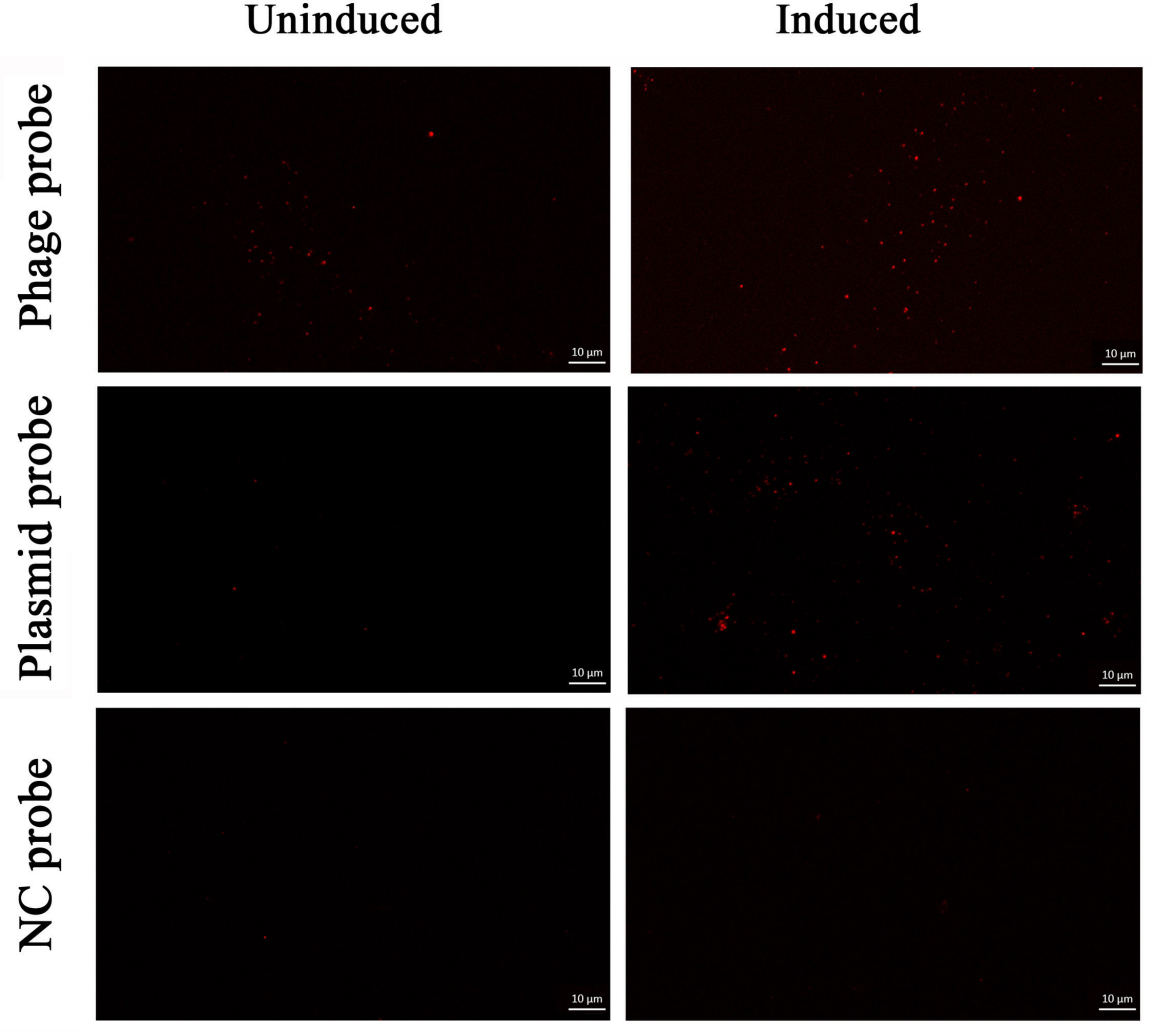
FISH of purified viral particles obtained from uninduced (left) and induced (right) *M. piezophila* cells using phage probes (top), plasmid probes (center), and negative control (NC) probes (bottom). The images show representative epifluorescence micrographs after FISH. Scale bars: 10 µm.

**Fig 6:**
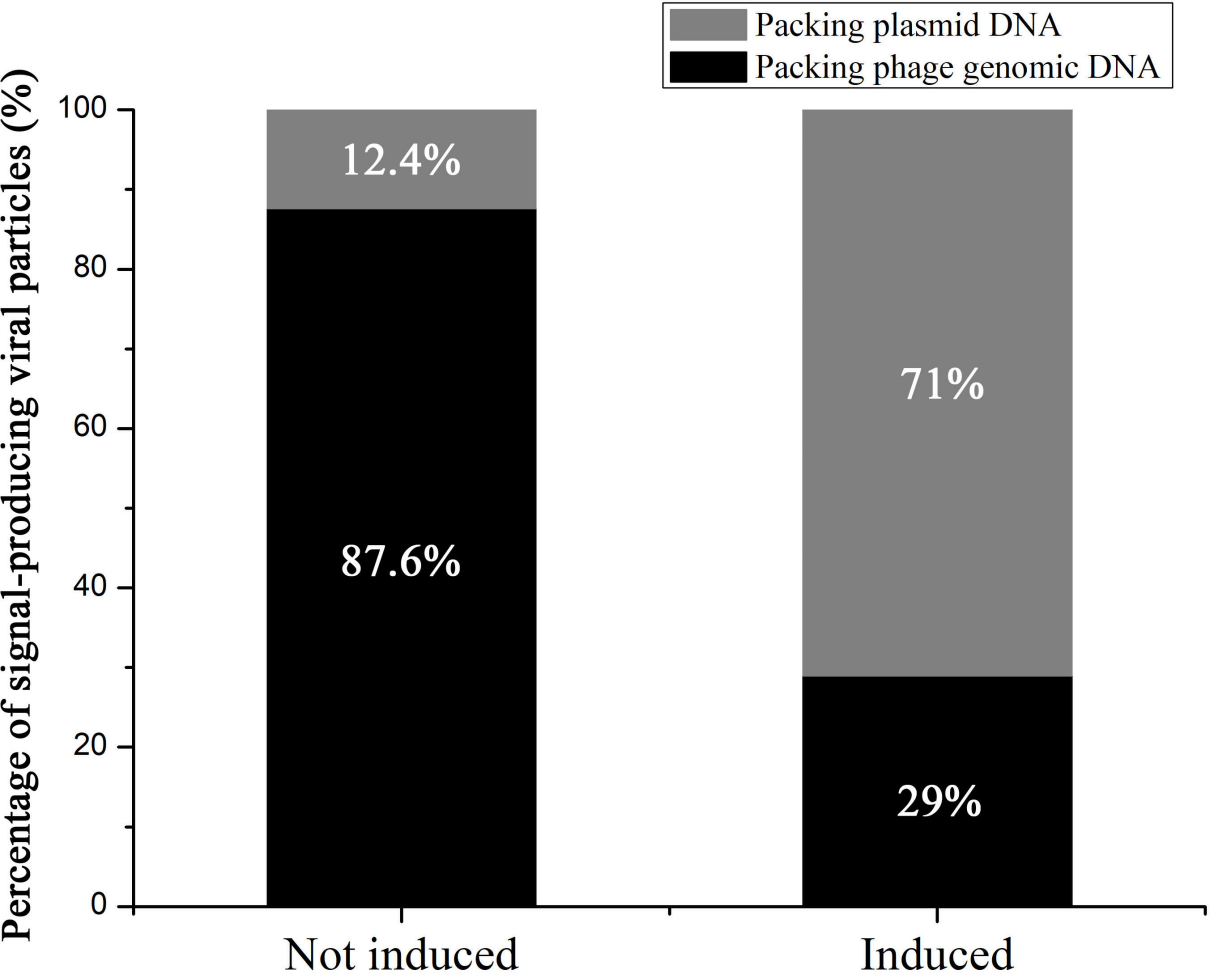
The percentage of signal-producing viral particles obtained from uninduced and induced *M. piezophila* cells that contain plasmid DNA and phage DNA as determined by FISH. The results are expressed as the means ± SD of three replicates.

To further verify our observations on the presence of pMP1 DNA in MPV1 phage particles and determine the plasmid:phage DNA ratio in the phage particles, FISH was conducted on purified virions obtained from induced and uninduced cells using phage and plasmid probes. The results revealed that the hybridized phage and plasmid probes emitted numerous (even a lot after induction) evenly distributed fluorescent dots with similar size (Fig. 5). In contrast, FISH of the purified induced and uninduced virion samples with NC probes showed few weak signals, which may be attributed to non-specific fluorescence or autofluorescence. These results indicate that the dot-like fluorescent signals emitted by phage and plasmid probes represent their specific hybridizations to phage and plasmid DNA, respectively, in the phage particles. In the uninduced group, only 12.4% of the signal-producing virion particles contain plasmid DNA (Fig. 5 and 6). However, the plasmid DNA is present in a greater number in mitomycin C induced virion particles, with approximately 71% of the signal-producing capsids containing plasmid DNA (Fig. 5 and 6). Therefore, induction led to a 2.4-fold (71% vs 29%) increase in the number of virions harboring plasmid signals than those harboring phage signals, consistent with our qPCR results, which showed 3-fold higher plasmid DNA than phage DNA in the induced *M. piezophila* cells (22). These results showed that both viral and plasmid DNA can be packaged into capsids even without induction, but plasmid DNA is preferentially packaged into capsids after induction.

## DISCUSSION

### First instance of molecular piracy identified in the deep-sea hydrothermal ecosystem

In this study, we used a modified phage-FISH to monitor the dynamics of phage–host–plasmid interactions at the single-cell level. Our results showed that a high ratio of phage capsids packed plasmid DNA instead of phage DNA, indicating that pMP1 hijacked MPV1 assembly machinery to facilitate its propagation and spread. Therefore, our study is the first to provide direct evidence of molecular piracy in the deep-sea hydrothermal ecosystem. Molecular piracy is a biological process, wherein MGEs (pirates) take advantage of other MGEs (helper) to promote their propagation and transfer (23). Some known examples of molecular piracy in prokaryotes are *Escherichia coli* P4/P2 system (31) and phage-inducible chromosomal islands (PICIs) (32). In the *E. coli* P4/P2 system, the P4 plasmid exploits the capsids of P2 phage to propagate and spread in *E. coli* cells (23, 31). PICIs are a family of pathogenicity islands in bacterial genomes, which manipulate the life cycle of certain temperate phages to promote their own spread. First identified in *Staphylococcus aureus*, PICIs are now thought to spread widely in both gram-positive and gram-negative bacteria (33).

Studies have demonstrated that pirates evolved a variety of strategies to manipulate the phage life cycle, involving complex interactions at several levels, from transcriptional control to macromolecular assembly (23, 34). For instance, these mechanisms include the redirection of bacteriophage capsid assembly and the specific recognition and packaging of MGE genomes into capsids (35). Some MGEs encode capsid protein homologs to redirect the capsid assembly pathway to generate smaller capsids that are more appropriate for packing MGEs rather than phage DNA (34, 36). However, in a precedent study, we did not observe smaller capsids or homologs of capsid proteins in pMP1, suggesting that pMP1 does not redirect the MPV1 capsid in the MPV1/pMP1 system (29).

PICIs have evolved two distinct phage packaging interference (Ppi) systems. *Staphylococcus aureus* pathogenicity islands (SaPIs), that employ a *pac* packing system, encode a Ppi protein which binds to the phage small terminase (TerS) and blocks phage packaging. Moreover, SaPIs encode TerS_S_ (a phage TerS homolog), which recognizes and packages SaPI DNA into the phage capsid (37). Contrastingly, some *E. coli* PICIs (EcPIs) that use a *cos* packing system express RppA (redirecting phage packaging protein), which blocks helper phage packaging but promotes EcPI DNA packaging upon binding with phage TerS (38, 39). In this study, we did not detect TerS homolog in pMP1 DNA, suggesting that pMP1 likely relies on phage TerS for plasmid DNA packaging. Furthermore, we did not detect any homologs of Ppi and RppA in pMP1 DNA, indicating that pMP1 likely employs a novel phage packaging interference system. Therefore, further investigations are required to explore the specific DNA packing strategy employed by pMP1. For instance, if pMP1 relies on phage TerS for DNA packaging, further analysis would be required to determine the mechanism by which phage TerS recognizes, cuts, and packages pMP1 DNA into capsids. Based on the sequence similarity between pMP1 DNA and MPV1 genome, we hypothesized that the strongest candidate for phage TerS recognition site is a 28 bp region located in Marpi_0300 of pMP1. In addition, it remains unknown how pMP1 could sense the induction of mitomycin C. We suspect that it may have a similar mechanism with the induction of temperate phages by mitomycin C, wherein mitomycin C activates the host SOS repair system by damaging host DNA (40).

### Implications of MPV1–pMP1 interplay in host–virus interactions

The interactions between plasmid and phage may have different implications for the fate of phage and host (41). For the host, carrying a prophage often confers resistance against related phages (42), whereas bearing a pirate MGE that interferes with phage production can enhance host cell survival (23). For example, several studies indicated that PICI-encoded mechanisms severely interfere with helper phage reproduction and thereby increase the survival of the bacterial host (37). Moreover, a recent study showed that a plasmid carried by *Klebsiella pneumoniae* could contribute to host defense against invasion by other transferable DNA elements at the cost of reduced host virulence (43). In contrast, pirates generally severely inhibit phage reproduction. Since some pirates (e.g., PICI) target conserved phage mechanisms, it is likely that these pirates act as the host’s innate immune system that the phages have to overcome to infect the host (34). However, not all pirate–helper phage interactions are antagonistic or competitive, and some may be based on cooperation or parasitism (44). For instance, Tormo-Mas et al. found that helper phages encode proteins that specifically induce PICI replication; however, the motive behind this evolutionary mechanism is unknown (45). Another study showed that a helper phage induces PICI to protect the phage and bacterial host from competing phages and other MGEs (46). A recent study identified a PICI family (cf-PICIs) that encodes all the proteins required for the production of small-sized capsids and the exclusive packaging of PICIs into these capsids; these PICIs only used the tails of the helper phage to propagate. Since these capsid-forming PICIs are not produced at the expense of helper phages, their relationship is not considered to be parasitic (47). These studies suggest that some pirates and their helper phages share a complex mutualistic relationship, which may affect the outcome of phage infection and HGT. Although the interplay between *M. piezophila* cells*,* MPV1, and pMP1 is not fully understood, our results suggest that pMP1 likely interacts with and modulates the viral machinery. Further studies on the MPV1/pMP1 system, especially on the mechanism by which MPV1 machinery recognizes and packages pMP1 DNA and mitomycin C induces pMP1 replication, are likely to advance our understanding of the plasmid-helper phage and host–MGE interactions that impact host–virus interactions and HGT in deep-sea vents. Since bacteria and phages are in an ongoing evolutionary “arms race” (48), we hypothesize that the resistance of MPV1 against pMP1-mediated molecular piracy will continue to evolve.

## Data Availability

The sequences of MPV1 genome and plasmid pMP1 are deposited in the GenBank database under accession numbers OZ018341.1 and CP003258.1, respectively.

## SUPPLEMENTAL MATERIAL

Supplemental material aem.02306-24-s0001.pdf Figure S1 and Table S1.

ASM does not own the copyrights to Supplemental Material that may be linked to, or accessed through, an article. The authors have granted ASM a non-exclusive, world-wide license to publish the Supplemental Material files. Please contact the corresponding author directly for reuse.
