# Supplementary material for: Molecular piracy in deep-sea hydrothermal vent: phage-plasmid interactions revealed by phage-FISH in Marinitoga piezophila
Source: Appl Environ Microbiol. 2025 Feb 27;91(3):e02306-24. doi: 10.1128/aem.02306-24 (PMC11921389; doi:10.1128/aem.02306-24)
Supplement: Supplemental material — Figure S1 and Table S1. [file aem.02306-24-s0001.pdf]

# Supporting Information

# Molecular Piracy in Deep-Sea Hydrothermal Vent: Phage-Plasmid Interactions revealed by Phage-FISH in *Marinitoga piezophila*

Min Jin[1,2,3†], Ouafae Rouxel[1,3†], Nadège Quintin[1,3], Claire Geslin[1,3*]

[1]Univ Brest, Ifremer, BEEP, Plouzané, 29280, France

[2]State Key Laboratory Breeding Base of Marine Genetic Resource, Third Institute of Oceanography, Ministry of Natural Resources, Xiamen, 361000, China

[3]LIA/IRP 1211 MicrobSea, Sino-French International Laboratory of Deep-Sea Microbiology, Plouzané, 29280, France

† These authors contributed equally to this work.

*Corresponding author (Claire Geslin): E-mail address: claire.geslin@univ-brest.fr

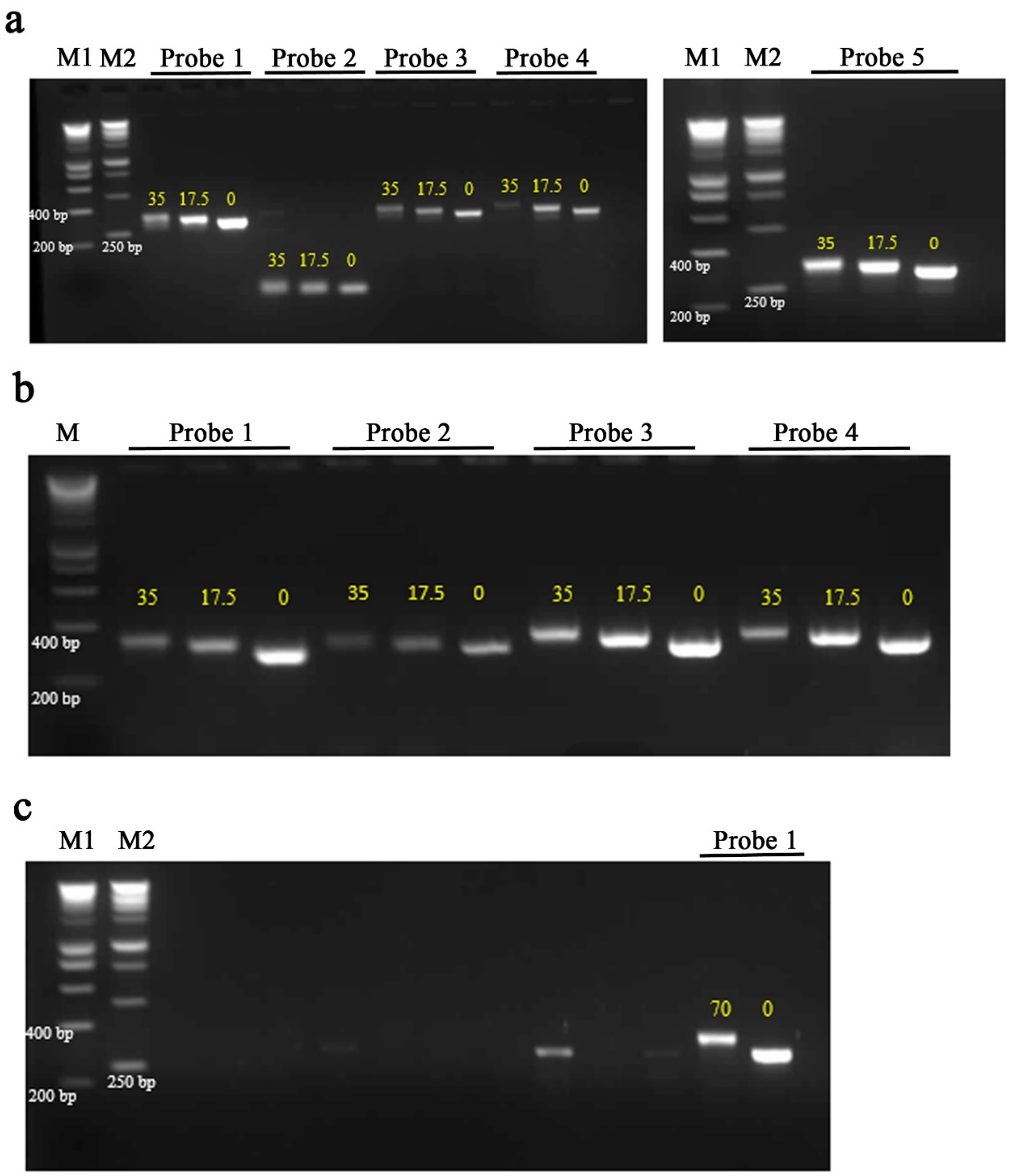


**Fig. S1 The PCR synthesis of DIG-phage probes (a), DIG-plasmid probes (b), and DIG-Negative probes (c).** The yellow text above the band indicates the final concentration of DIG (μM) used for PCR. The white text indicates the length of DNA marker. M, M1, and M2 indicate DNA markers. The primers used for PCR are shown in Table 1.

**Table S1 dsDNA Polynucleotide probes (300 bp each) used in this study**

| Probes | Template | Probe number | Targeted Region | %GC | Forward Primer | Reverse Primer |
|---|---|---|---|---|---|---|
| Phage Probe | Phage genome DNA | Probe 1 | 34141-34440 bp | 29.7 | GAATTTATAAGCCCAGATGATATTG | AAATGGAGAGAAAAAATTCAAGCGT |
| | | Probe 2 | 34441-34740 bp | 24.7 | TTTCTTTAATCTTTCAGGTGTTTCC | AGGTTATTGGAATTTGATTTTGACG |
| | | Probe 3 | 34741-35040 bp | 32.3 | ATGCTTAACGTGTAAAAATACTTCA | AGGAAAAGATCATCAGAATTGA |
| | | Probe 4 | 35041-35340 bp | 32 | ACTTCTCTTATTACCTTTTCCA | TCAAAAAAATACTGGATGGGT |
| | | Probe 5 | 35341-35640 bp | 33 | GGCAATATCTTTCATTCTCAT | GACTCTGGAAAGAAAATGAAA |
| Plasmid Probe | Plasmid DNA | Probe 1 | 7981-8280 bp | 27.3 | GATGTGATCATAGATTTTATTAATC | ATTAGCCTTCTTAACTTTTTAG |
| | | Probe 2 | 8281-8580 bp | 30 | CCGTCGTTTCAACGGGAGAGCT | CAAAATTTATGTAATATTGTATATC |
| | | Probe 3 | 9181-9480 bp | 27 | TATTCATTTAAGGGGTTTAAG | AACATAACTTTCATCATCCA |
| | | Probe 4 | 9781-10080 bp | 33.7 | TTTATAATGTAGGTATAAACTACACTG | TAGTTAGGCCATCTTTACCAAG |
| Negative probe | *Thermococcus thioreducens* genome DNA | Probe 1 | 1745340-1745639 bp | 59.4 | CCGGAATACTCCTCGGTTTCCT | TGAATTTACCCGCGTAGATTCTC |
